# Doxorubicin Loaded PLGA Nanoparticle with Cationic/Anionic Polyelectrolyte Decoration: Characterization, and Its Therapeutic Potency

**DOI:** 10.3390/polym13050693

**Published:** 2021-02-25

**Authors:** Li-Hui Tsai, Chia-Hsiang Yen, Hao-Ying Hsieh, Tai-Horng Young

**Affiliations:** 1 Department of Biomedical Engineering, National Taiwan University, Taipei 100, Taiwan; d07528009@ntu.edu.tw (L.-H.T.); f04548042@ntu.edu.tw (C.-H.Y.); Haoying@ntu.edu.tw (H.-Y.H.); 2 Department of Dentistry, National Taiwan University Hospital, Taipei 100, Taiwan; 3 Department of Biomedical Engineering, National Taiwan University Hospital, Taipei 100, Taiwan

**Keywords:** doxorubicin, carboxylic acid terminated PLGA, ester terminated PLGA, polyethylenimine, poly(acrylic acid), endo/lysosomal escape, pH-dependent

## Abstract

Optimized Doxorubicin hydrochloride (DOX) loaded poly(lactic-co-glycolic acid) (PLGA) nanoparticles (DPN) were prepared by controlling the water/oil distribution of DOX at different pH solutions and controlling the electrostatic interaction between DOX and different terminated-end PLGAs. Furthermore, cationic polyethylenimine (PEI) and anionic poly (acrylic acid) (PAA) were alternately deposited on DPN surface to form PEI-DPN (IDPN) and PAA-PEI-DPN (AIDPN) to enhance cancer therapy potency. Compared to DPN, IDPN exhibited a slower release rate in physiological conditions but PEI was demonstrated to increase the efficiency of cellular uptake and endo/lysosomal escape ability. AIDPN, with the outermost negatively charged PAA layer, still retained better endo/lysosomal escape ability compared to DPN. In addition, AIDPN exhibited the best pH-dependent release profile with 1.6 times higher drug release in pH 5.5 than in pH 7.4. Therefore, AIDPN with the characteristics of PEI and PAA simultaneously was the most optional cancer therapy choice within these three PLGA nanoparticles. As the proposed nanoparticles integrated optimal procedure factors, and possessed cationic and anionic outlayer, our drug delivery nanoparticles can provide an alternative solution to current drug delivery technologies.

## 1. Introduction

Nanomedicine [1] plays an important role in cancer therapy because it can accumulate in tumors with a high vessel density, surrounded by an enhanced permeability and retention (EPR) effect [2,3]. Compared to the fast elimination of free drugs, nanomedicine exhibits a sustained release of up to four weeks [4,5]. Recently, many materials have been used to make drug delivery nanoparticles [6]. These materials should demonstrate biocompatibility, biodegradability, and low toxicity [7,8]. Poly(lactic-co-glycolic acid) (PLGA) is one of the most successful polymers used in the drug delivery system and is approved by the Food and Drug Administration (FDA) and the European Medicines Agency (EMA) in drug delivery systems [9,10]. Depending on the properties of drugs, preparation methods such as emulsification–evaporation, nanoprecipitation, spontaneous emulsion solvent diffusion, and membrane emulsification, have been chosen to produce drug-loaded PLGA nanoparticles [11,12].

Doxorubicin hydrochloride (DOX), an anthracycline anticancer drug, is used widely in several human cancers by DNA damage through two proposed mechanisms, which are topoisomerase II inhibition and free radical generation [13,14]. However, dose-limited cardiotoxicity and myelosuppression restrict DOX treatment in cancer therapy [15,16]. Antitumor drugs encapsulated in biodegradable polymers is one of the successful methods used to reduce their side effects [17,18].

The purpose of this study is to systematically analyze and optimize the effect of various variables on the size, zeta potential, and encapsulated efficiency percentage (EE%) of drug-loaded PLGA nanoparticles. In general, PLGA nanoparticles are deficient in the endo/lysosomes escape capability. Therefore, polyethylenimine (PEI), with the “proton sponge’’ effect rupture endo/lysosomes [19,20,21,22] was employed in the system by direct addition of PEI to nanoparticle formulation and by coating on PLGA nanoparticle surface. However, the positive charge of PEI is potentially cytotoxic and is tended to aggregate with serum protein [23,24]. To diminish this defect of PEI, another anionic polyelectrolyte poly(acrylic acid) (PAA) was involved in this drug-loaded nanomedicine, which could be adsorbed on PEI because of its anionic polyelectrolyte property [25].

In the present study, DOX-loaded PLGA nanoparticle (DPN), PEI-DPN (IDPN), and PAA-PEI-DPN (AIDPN) were prepared. Characterization of these nanoparticles, including particle size, zeta potential, morphology on transmission electron microscopy (TEM), and DOX release profile were determined. Cell cytotoxicity, cellular uptake, and endo/lysosomes escape of DPN, IDPN, and AIDPN were analyzed using A549, non-small cell lung cancer (NSCLC) cell line. A549 was chosen as the cell testing model because lung cancer is the leading cancer-related cause of death, and NSCLC is the most common type of lung cancer [26,27]. Finally, nanoparticles decorated with the combination of cationic/anionic polyelectrolyte (PEI/PAA) were found to be the most suitable in cancer therapy.

## 2. Materials and Methods

### 2.1. Materials

PLGA (carboxylic acid terminated, Mw = 7000–17,000 Da; ester terminated, Mw = 30,000–60,000 Da), polyvinyl alcohol (PVA, Mw = 13,000–23,000 Da), dichloromethane (DCM), polyethylenimine (PEI, Mw = 25,000 Da), poly(acrylic acid) (PAA, Mw = 15,000 Da), dimethyl sulfoxide (DMSO), and 3-(4,5-dimethylthiazol-2-yl)-2,5-diphenyltetrazolium bromide (MTT) were purchased from Sigma-Aldrich (St. Louis, MO, USA). Doxorubicin hydrochloride (DOX) was purchased from LC Laboratories (Woburn, MA, USA). Ultrapure water (18.2 MΩ·cm at 25 °C) was prepared by Cascada Purification System (Washington, NY, USA).

### 2.2. Preparation of DOX Loaded PLGA Nanoparticles

DOX-loaded PLGA nanoparticles were prepared using the double emulsion (W1/O/W2) method. Briefly, 0.5% DOX aqueous solution (W1) was added to 2% carboxylic acid terminated PLGA (A-PLGA) or ester terminated PLGA (E-PLGA) in DCM (oil phase; O) with a 1:5 volume ratio and sonicated in 80% amplitude for 60 s to emulsify W1-in-O (W1/O). Then, the primary W1/O was poured into the 5 mL outer water (W2), which was composed of 1% PVA in water or 1% PVA in phosphate buffer (PB) at pH 8 and sonicated for another 60 s in 50% amplitude. To evaporate DCM, W1/O/W2 solution was stirred at 20 °C overnight. A-PLGA/water, A-PLGA/PB, E-PLGA/water, and E-PLGA/PB were centrifuged by 22,140× *g* for 30 min and washed twice with water. DPN represented the formulation of A-PLGA/PB. DPN was than resuspended in waster and stored at 4 °C before use.

### 2.3. Preparation of iDPN and i’DPN

PEI was incorporated in DPN by adding 2 mg PEI in W1 (i) or O (i’) during the primary emulsification process. Primary W1/O was then poured into 1% PVA in water or 1% PVA in pH 8 PB W2 to produce i/water-DPN, i/PB-DPN, i’/water-DPN, and i’/PB-DPN. All other processes were the same as previously mentioned.

### 2.4. Preparation of IDPN and AIDPN

Layer by layer (LBL) DPN was prepared by electrostatic interaction. To prepare positively charged IDPN, negatively charged DPN was added into 0.2% PEI (pH 7), incubated for 30 min, and then centrifuged by 22,140× *g* for 10 min twice to remove excess PEI. To prepare negatively charged AIDPN, condensed IDPN was added dropwise into gently stirring 0.2% PAA (pH 7) for another 30 min incubation. Excess PAA was removed by 22,140× *g* for 10 min centrifugation. Then, the palette of nanoparticles was resuspended in water and centrifuged by 22,140× *g* for another 10 min.

### 2.5. Characterization of Nanocarriers

DPN, IDPN, and AIDPN were suspended in water, and particle size and zeta potential were measured using ZETASIZER (Malvern Nano-ZS90, Malvern, Worcestershire, UK); DPN, IDPN, and AIDPN were diluted in water to 150–200 kcps of ZETASIZER detection. Morphology of nanoparticles (100,000× magnification) was observed by TEM (Hitachi H-700 Transmission Electron Microscope, Tokyo, Japan); nanoparticles were dropped on carbon-coated copper grids (EM REsolution, Sheffield, UK).

### 2.6. Encapsulation Efficiency

To determine the encapsulated efficiency percentage (EE%), nanoparticles were added to DMSO and sonicated for 10 min. After PLGA was completely dissolved in DMSO, the DOX content was measured by UV–visible spectrophotometer at 480 nm. The EE% of each formulation was calculated by the following equation:

(1) $$\mathrm{EE}\%=\left[\;\frac{\mathrm{amount}\;\mathrm{of}\;\mathrm{DOX}\;\mathrm{in}\;\mathrm{nanoparticles}\;}{\mathrm{amount}\;\mathrm{of}\;\mathrm{DOX}\;\mathrm{initial}\;\mathrm{added}}\right]*100\%$$

### 2.7. In Vitro Release Profile of DOX from DPN, IDPN, and AIDPN

A quantity of 60 μg of DOX contained in DPN, IDPN, and AIDPN were respectively suspended in 5 mL PBS (pH 7.4 or pH 5.5, 37 °C) with constant shaking at 100 rpm for 3 days. At each defined time point, nanoparticle suspensions were centrifuged at 22,140× *g* for 30 min and 1 mL of supernatant was replaced with PBS (pH 7.4 or pH 5.5). DOX in the supernatant was determined by the measurement of fluorescence (Ex:480/Em:550).

### 2.8. Cell Culture

A549, a human non-small cell lung cancer cell line (NSCLC), obtained from BCRC (Bioresource Collection and Research Center, Hsinchu, Taiwan), was grown in F-12K Medium (Kaighn’s Modification of Ham’s F-12 Medium) (Gibco^®^, Gaithersburg, MD, USA), supplemented with 10% fetal bovine serum (FBS; Biological Industries, Cromwell, CT, USA) and 1% antibiotic–antimycotic (Gibco^®^, Gaithersburg, MD, USA). A549 was incubated at 37 °C in a 5% CO_2_ humidified atmosphere.

### 2.9. Cytotoxicity

MTT assay was used to measure the cell viability after A549 was incubated with free DOX, DPN, IDPN, and AIDPN. Briefly, A549 was seeded (3500 cells/well) in 96-well plates. After overnight pre-incubation, free DOX, DPN, IDPN, and AIDPN from 0 to 1 μM were added. Cytotoxicity test in 24 h treatment, cells were incubated in a free DOX/nanoparticles-filled medium for 24 h. Then, cells were washed twice with PBS and were incubated in fresh medium for another 24 h. Cytotoxicity test in 48 h treatment, cells were incubated in a free DOX/nanoparticles-filled medium for 48 h. After incubation, the medium was then removed, and 100 μL of 5 mg/mL MTT was added and incubated for 4 h at 37 °C. Finally, the supernatant was removed, and 100 μL DMSO was added to dissolved formazan. The viability of A549 was evaluated by the absorbance of 570 nm.

### 2.10. Cellular Uptake

The uptake of free DOX, DPN, IDPN, and AIDPN was determined using flow cytometry FACSVerseTM (BD Biosciences, San Jose, CA, USA). A549 was seeded (10^5^ cells/well) in 6-well plates at 37 °C. After overnight pre-incubation, 0.1 and 1 μM of free DOX, DPN, IDPN, and AIDPN were added and incubated for another 24 h and 48 h. A549 grown in medium without free DOX or nanoparticles was used as a control group. Then, A549 cells were washed with PBS and resuspended in 800 μL PBS. A549 cells (10^4^ cells/experiment) were analyzed by flow cytometry, and the fluorescence was detected by PE wavelength. The mean fluorescence intensity (MFI) was acquired by BD FACSuite software (BD Biosciences, San Jose, CA, USA).

### 2.11. Localization of Free DOX, DPN, IDPN, and AIDPN in Cells

A549 was seeded (2 × 10^4^ cells/well) in 24-well plates and incubated overnight at 37 °C. Then, 1 μM of free DOX, DPN, IDPN, and AIDPN was added and incubated for another 24 hr. After incubation, A549 was washed with PBS twice and stained with Hoechst 33342 and Lysotracker green DND-26. The fluorescence image of DOX, nuclei, and endo/lysosomes were obtained using a fluorescent microscope (Leica, Wetzlar, Germany).

### 2.12. Statistical Analysis

Data are represented as the mean ± standard deviation (S.D.) of three repeat experiments. Student’s *t*-test and one-way ANOVA with post hoc tests were used to compare experimental data, considering *p* < 0.05 as statistically significant.

## 3. Results

### 3.1. Characterization of DPN

**Figure 1:**
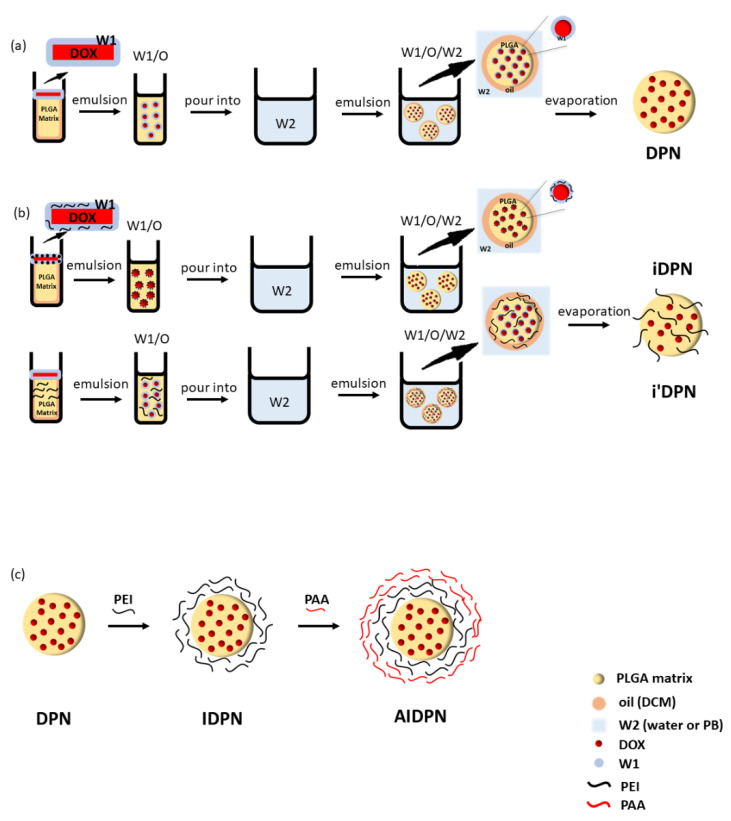
Schematic representation of (**a**) DPN (DOX-loaded PLGA nanoparticle), (**b**) iDPN (PEI was added in W1 during the incorporation process), i’DPN (PEI was added in O during the incorporation process), (**c**) IDPN (PEI-DPN), and AIDPN (PAA-PEI-DPN). Abbreviations: DCM, dichloromethane; DOX, Doxorubicin hydrochloride; W1, 0.5% DOX aqueous solution; W2, outer water, which was water or PB (phosphate buffer; pH 8); PEI, polyethylenimine; PAA, poly(acrylic acid).

**Figure 2:**
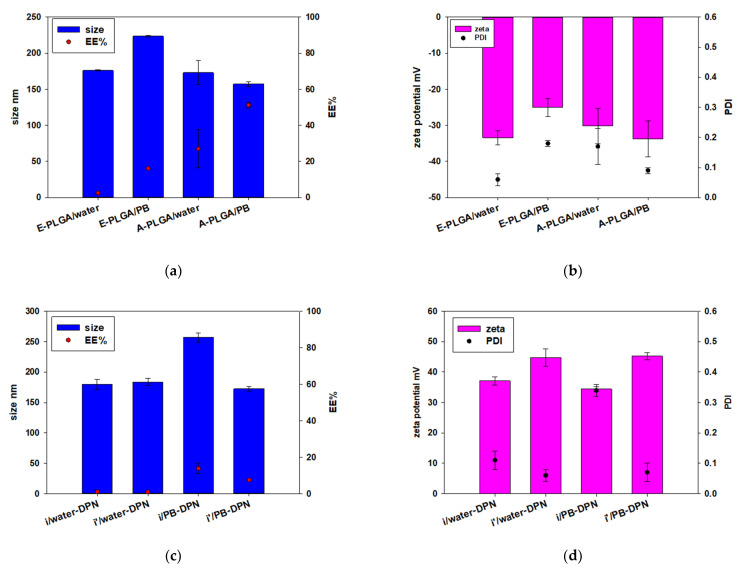
The effect of different types of PLGA and W2 on (**a**) diameter and EE% (encapsulated efficiency percentage) (**b**) zeta potential and PDI (polydispersity index) of DPN, and the effect of incorporated PEI and W2 on (**c**) diameter and EE% (**d**) zeta potential and PDI of iDPN and i’DPN. (n = 3; mean ± standard deviation). E-PLGA/water (E-PLGA was dissolved in O and W2 was water), E-PLGA/PB (E-PLGA was dissolved in O and W2 was PB), A-PLGA/water (A-PLGA was dissolved in O and W2 was water), A-PLGA/PB (A-PLGA was dissolved in O and W2 was PB), i/water-DPN (PEI was added in W1 during the incorporation process and W2 was water), i’/water-DPN (PEI was added in O during the incorporation process and W2 was water), i/PB-DPN (PEI was added in W1 during the incorporation process and W2 was PB), i’/PB-DPN (PEI was added in O during the incorporation process and W2 was PB). Abbreviations: E-PLGA, ester terminated PLGA; A-PLGA, carboxylic acid terminated PLGA; PB, phosphate buffer (pH 8).

Because DOX has high solubility in aqueous solution, the present study used double emulsion to encapsulate DOX in PLGA nanoparticles (Figure 1a) [28,29]. The EE% of two different types of PLGA (A-PLGA and E-PLGA) and two different types of W2 (water and PB; pH 8) were compared in this study. Figure 2a shows E-PLGA had a lower EE% than A-PLGA regardless of whether W2 was water or PB. In addition, W2 composed of PB increased EE% compared to water in both A-PLGA and E-PLGA. The size of all formulations was around 150 to 230 nm, and zeta potential was around −25 mV to −35 mV (Figure 2a,b). A-PLGA/PB was the best formulation due to its highest EE%. DPN for later experiments was prepared using the A-PLGA/PB formulation.

### 3.2. DPN Decorated with Polyelectrolytes

In addition, polycationic PEI was used to change the zeta potential of DPN to a positive value by the incorporation method (Figure 1b) [30,31] and adsorption method (Figure 1c). However, when PEI was incorporated in DPN by adding it in the W1 (iDPN) phase or the O (i’DPN) through the double emulsion process, the EE% was relatively low, regardless of whether water or PB was W2 (Figure 2c). Each of these four types of DPN incorporated by PEI exhibited a positive zeta potential (Figure 2d).

**Figure 3:**
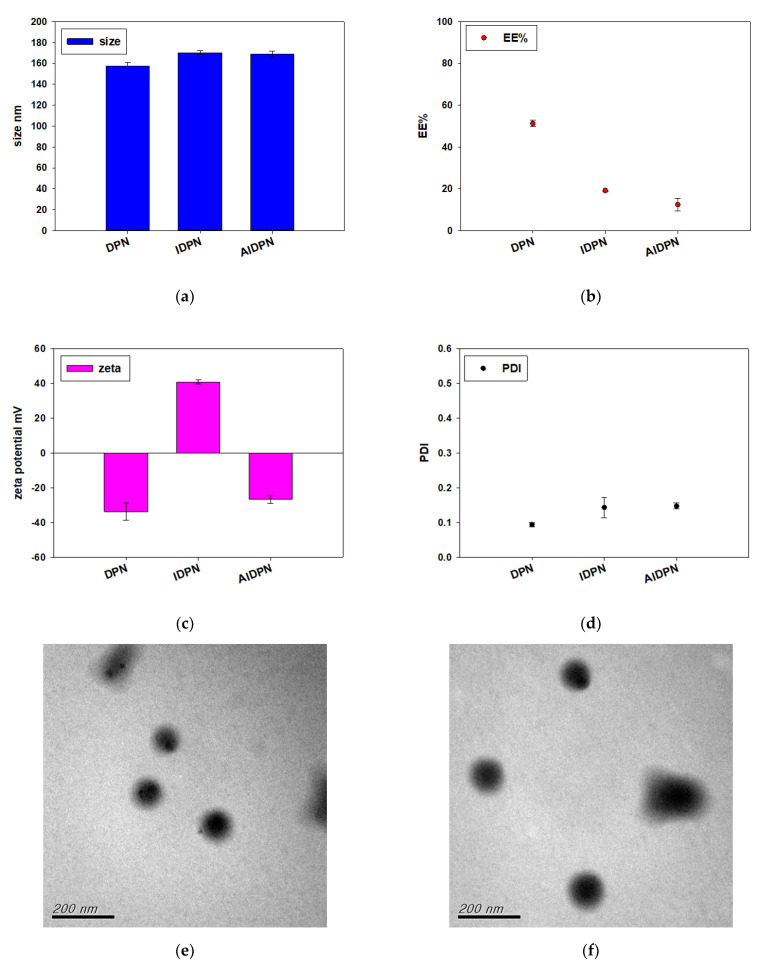

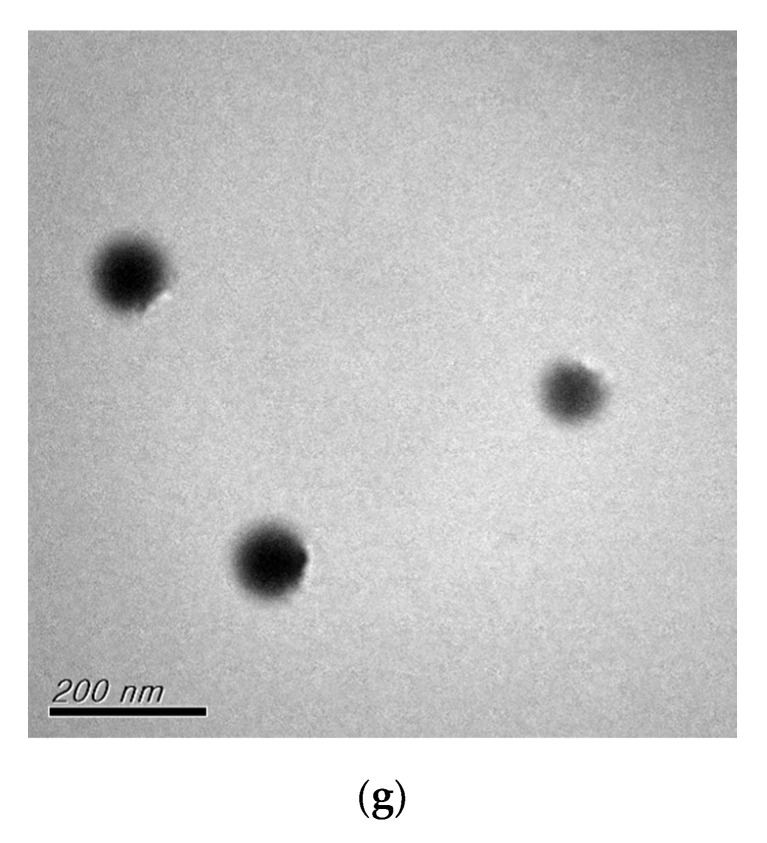
Characterization of DPN, IDPN, and AIDPN. (**a**) diameter, (**b**) EE%, (**c**) zeta potential, and (**d**) PDI. (n = 3; mean ± standard deviation). Morphology of (**e**) DPN, (**f**) IDPN, and (**g**) AIDPN on TEM image.

Figure 3 shows the variation of size, EE%, zeta potential, and PDI of DPN surface coated by PEI (IDPN), and then by PAA (AIDPN). After the deposition of PEI and PAA onto the core DPN surface, the particle size increased slightly and the surface charge changed from −33.77 ± 4.97 mV to 40.74 ± 1.18 mV for IDPN and −26.76 ± 2.20 mV for AIDPN, which confirmed the expected charge reversal and the opposite charge polycation/polyanion absorption. Similar to results of the incorporation method, the EE% decreased when PEI and PAA were assembled onto the DPN surface, which may be attributed to the additional washing and centrifugation steps. TEM photographs show the morphology of DPN, IDPN, and AIDPN was round (Figure 3e–g).

### 3.3. The Release Profile of DPN, IDPN, and AIDPN

**Figure 4:**
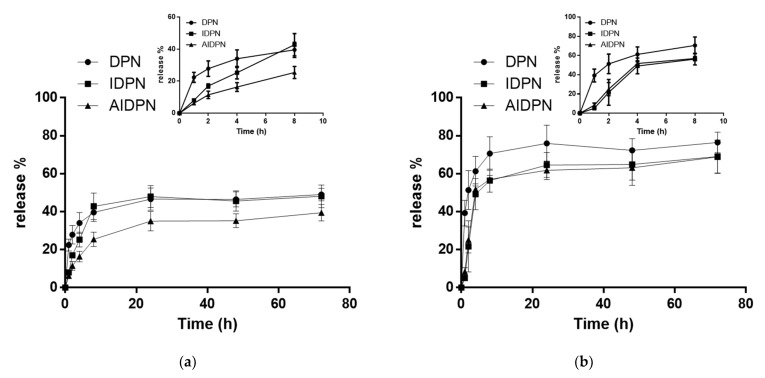
The release profile of DPN, IDPN, and AIDPN in (**a**) pH 7.4 PBS and (**b**) pH 5.5 PBS at 37 °C within 3 days. Inserted graphs show the release profile of DPN, IDPN, and AIDPN within 8 h in pH 7.4 PBS and pH 5.5 PBS. (n = 3; mean ± standard deviation).

The controlled release profile of DOX was successfully characterized by using PLGA as a carrier of nanomedicine at both pH 7.4 or pH 5.5 PBS solution (Figure 4). All three types of nanoparticles DPN, IDPN, and AIDPN at pH 5.5 had higher release rate than at pH 7.4 within three days. DPN had the highest initial release rate at both pH conditions. At pH 7.4, DOX release from AIDPN was less than that from DPN and IDPN. From the inserted graph of Figure 4, during the first eight hours at pH 7.4, there was a significant difference in the release slope (release%/hour) between IDPN and AIDPN (*p* < 0.05). However, a significant difference between IDPN and AIDPN was not observed at pH 5.5.

### 3.4. Cytotoxicity

**Figure 5:**
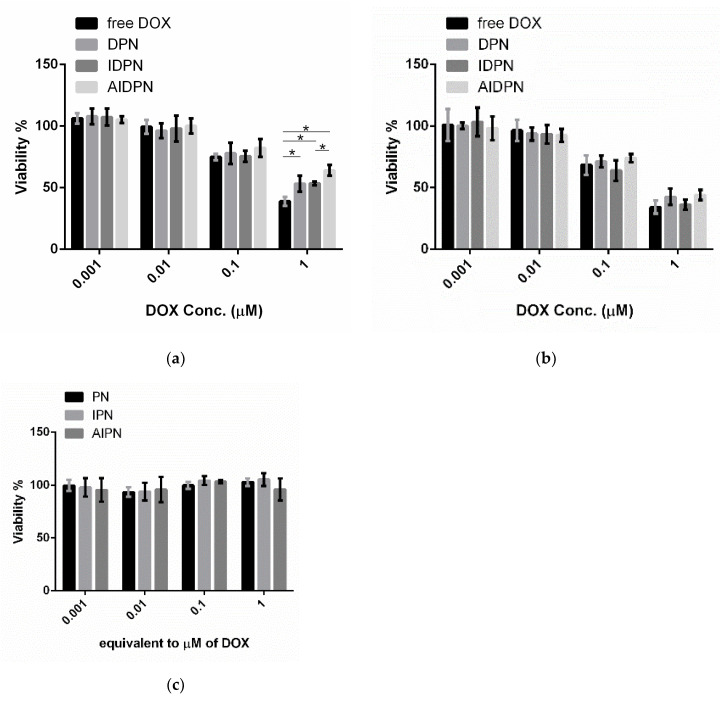
Cytotoxicity of free DOX, DPN, IDPN, and AIDPN with different concentrations for (**a**) 24 h and (**b**) 48 h incubation, and (**c**) blank nanoparticles for 48 h incubation in A549. * indicates comparison of two group, *p* < 0.05.

After treatment of DPN, IDPN, and AIDPN for 24 and 48 h, the viability of A549 cells decreased with increasing concentration (Figure 5). After treatment for 24 h, the difference of cytotoxicity of nanoparticles only could be observed in a 1 μM concentration. Free DOX showed the highest cytotoxicity in this study and IDPN had a significantly higher cytotoxic effect than AIDPN (*p* < 0.05). However, all three nanoparticles showed similar results after treatment for 48 h. These nanoparticles without DOX had no toxicity to A549 cells for 48 h of incubation (Figure 5c).

### 3.5. Cellular Uptake

**Figure 6:**
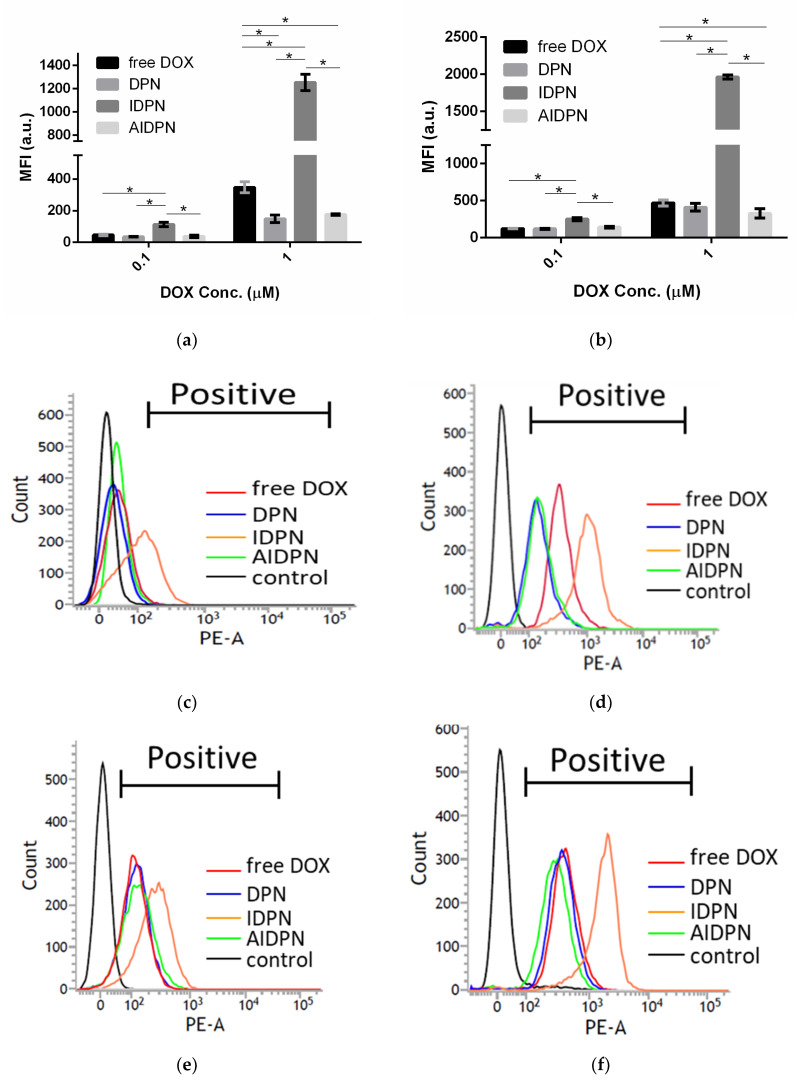
MFI in A549 after 0.1 and 1 μM of free DOX, DPN, IDPN and AIDPN for (**a**) 24 h and (**b**) 48 h incubation. Fluorescence intensity in A549 measured by flow cytometry after 24 h incubation of (**c**) 0.1 μM and (**d**) 1 μM of free DOX (red), DPN (blue), IDPN (orange), AIDPN (green), and control (black) and 48 h incubation of (**e**) 0.1 μM and (**f**) 1 μM of free DOX (red), DPN (blue), IDPN (orange), AIDPN (green), and control (black). Abbreviations: MFI, mean fluorescence intensity. * indicates comparison of two group, *p* < 0.05.

Cellular uptake of free DOX, DPN, IDPN, and AIDPN was determined by the fluorescence intensity of DOX in A549 cells. Both the mean fluorescence intensity (MFI) and the number of positive cells increased with increasing nanoparticle concentration and incubation time (Figure 6). As expected, the highest uptake of IDPN nanoparticles with positive surface charge was observed in A549 cells at all incubation conditions. In contrast, although both DPN and AIDPN with negative surface charge could be taken up by A549 cells, their MFI was not higher than that of free DOX.

### 3.6. Endo/Lysosomes Escape Efficiency of DPN, IDPN, and AIDPN

**Figure 7:**
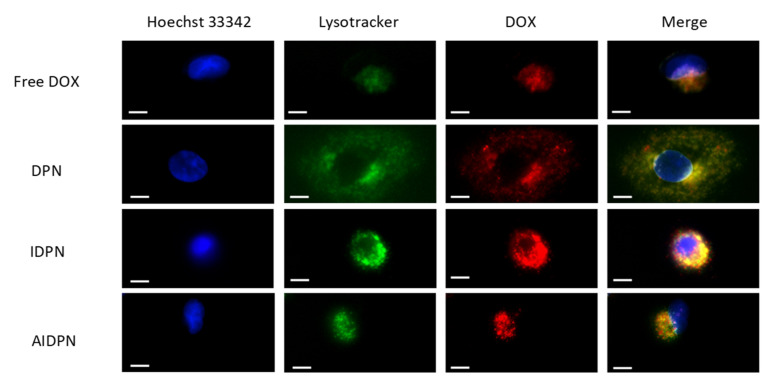
Fluorescence image of free DOX, DPN, IDPN, and AIDPN in A549. Blue represented nucleus (stained with Hoechst 33342); green represented endo/lysosomes (stained with Lysotracker); red was the DOX fluorescence. Scale bar 10 µm.

The colocalization (yellow) of DOX (red) and Lysotracker (green) indicated that DPN, IDPN, and AIDPN were internalized in endo/lysosomes (Figure 7). IDPN was most capable of endo/lysosomal escape because it showed the greatest amount of separation of red and yellow colors. The rank of the ability of endo/lysosomal escape from high to low was IDPN, AIDPN, and DPN. Free DOX was also sequestered in acidic compartments, but it did not find that any free DOX escaped.

## 4. Discussion

In this study, DOX was loaded in PLGA nanoparticles for controlling release. To optimize the formulation of DPN, two types of PLGA with carboxyl and ester end groups, and two types of PVA-containing W2, water (pH 5–6), and PB (pH 7–8), were compared. According to Figure 2a, the ideal formulation was composed of A-PLGA and PB. DOX acts as a cationic ion at a pH around 4–6 and a less hydrophilic neutral compound at pH around 8.2 because the amine group pKa of DOX is 8.2 [32,33]. Therefore, the optimal formulation is probably explained by the stronger electrostatic interaction of cationic DOX and anionic A-PLGA in a weakly acidic environment, and less hydrophilic DOX is distributed to the oil phase in W2 composed of PB in a weakly basic environment during the nanoparticle formation process.

Recently, because PEI has been used in gene delivery systems due to its transfection efficiency [34,35], PEI was further added to the DPN formulation to enhance the therapeutic potency. Two procedures of PEI employed in DPN were compared in this study: (1) direct addition of PEI to nanoparticle formulation by single procedure; and (2) PEI decoration on the DPN surface using the electrostatic absorption process. However, positively charged PEI could occupy the carboxylic acid of A-PLGA, so both A-PLGA and E-PLGA had low efficiency to encapsulate DOX in this situation (Figure 2c). Therefore, the DPN surface was decorated through an additional layer of PEI. In addition, PAA, pH-dependent anionic polyelectrolyte, has been used to prepare a pH-responsive drug delivery system [36,37]. To prepare DOX-loaded nanoparticles with both endo/lysosomal escape and pH sensitive property, the present study tried to decorate PEI and PAA on DPN using the LBL self-assembly technique.

During the LBL self-assembly process, the pH value of polyelectrolyte solutions should be considered. The ionization degree of PAA was increased with the increasing pH value, and the protonated degree of PEI could be enhanced with the decreasing pH value [38,39]. Furthermore, both alkaline and acid surroundings accelerated the degradation of PLGA [40]. Due to all of these restrictions, the process of LBL self-assembly should be applied using a solution of around pH 7.

Due to the penetration of water in nanoparticles enhanced by the high content of water-soluble drugs [41], hydrophilic DOX in DPN might facilitate PBS penetration into DPN. DOX was then transported into the water-filled pore network and diffused into the medium. Again, because acid surrounding could increase the solubility of cationic DOX, the amount of DOX in pores would increase when DPN was suspended in pH 5.5 PBS. As a result, all DPN, IDPN, and AIDPN had a higher release percentage at pH 5.5 PBS than at pH 7.4 PBS (Figure 4). Generally, the environment surrounded tumor tissue is slightly acidic than physiological conditions. This release profile implied that the acidic condition can promote DOX released from DPN, IDPN, and AIDPN, with the potential for selective tumor therapy. In particular, DOX release of AIDPN at pH 5.5 continued to constantly increase within the 72 h of the experiment until it reached its equilibrium at 63.9%, showing a significantly higher drug release (39.4%) than the corresponding nanoparticles in the physiological solution (pH 7.4). The increased DOX release at pH = 5.5 can be attributed to the higher anionic degree of PAA at pH 7.4 than at pH 5.5 [38,39] with stronger binding affinity to protonated DOX, which resulted in an approximately 1.6 times higher drug release from AIDPN at pH = 5.5 compared to that at pH = 7.4. Due to the additional PAA layer, the release profile of IDPN and AIDPN was significantly different in pH 7.4 PBS but similar in pH 5.5 PBS (Figure 4). Therefore, AIDPN can be considered the best option in this study with the lowest DOX release from nanoparticles to reduce the side effect on normal cells under physiological conditions.

Previous research has demonstrated that the uptake of nanoparticles with a positive surface charge was higher than that with a negative surface charge in cancer cells [42]. Similarly, IDPN had significantly higher efficiency than DPN and AIDPN in cellular uptake (Figure 6). However, compared to that of DPN, the higher uptake of IDPN by A549 cells did not correspond with the higher cytotoxicity at 1 μM concentration (Figure 5), which may be due to the more DOX release from DPN within the initial four hours (Figure 4). This indicates the burst effect of DPN could be shielded by the PEI layer on the outside surface. Additionally, the PAA layer on the IDPN surface further shielded the AIDPN, possibly contributing to low release and cytotoxicity.

The endo/lysosomal escape of drug is considered to affect the efficiency of drug cytotoxicity strongly [43]. IDPN was shown to be the most efficient endo/lysosomes escape in three different types of nanoparticles (Figure 7). This implied that IDPN was entrapped in cells by endocytosis and would be delivered to cytoplasm rather than degraded in acidic endo/lysosomes or ejected by lysosome secretion [44,45]. The escape ability of IDPN could be attributed to PEI, whose buffer capacity was supposed to rupture acid organelles by the “proton sponge’’ effect [19,20,21,22]. PAA seemed to diminish the escape ability contributed from PEI due to fewer red spots spread from the yellow region when A549 was incubated with AIDPN. However, compared to DPN, AIDPN, with the outermost negatively charged PAA layer, still retained better endo/lysosomal escape ability. Therefore, the cationic/anionic LBL controlled release nanoparticle, AIDPN, is an ideal formulation in cancer therapy for its pH-dependent release profile and endo/lysosomal escape ability. In further investigation, we will deliver AIDPN to animal model to evaluate safety, efficacy, and pharmacokinetics in vivo. Furthermore, we plan to prepare a lyophilized formulation of AIDPN to dissolve the problem of not-convenient for commercial application.

## 5. Conclusions

In summary, based on the optimized formulation of A-PLGA and the W2 phase, three types of nanoparticles—DPN, IDPN, and AIDPN—were prepared using the PEI/PAA LBL technique. DPN showed poor ability in terms of endo/lysosomal escape, and its undesired high burst release in physiological conditions might result in greater toxicity in normal cells. IDPN decorated with PEI, compared to DPN, had a slower release rate in physiological conditions but had higher efficiency in cellular uptake and endo/lysosomal escape. However, the cationic surface of IDPN might restrict its application in vivo due to aggregation with serum protein. AIDPN, with the outermost negatively charged PAA layer, retained endo/lysosomal escape ability and mitigated the risk of aggregation with serum protein in vivo. In addition, the pH-sensitive PAA modified AIDPN to possess the best pH-dependent release profile. As a result, AIDPN, with the properties of both PEI and PAA, was found to be the most suitable cancer therapy amount these three different nanoparticles.

## Data Availability

Not applicable.

## Abbreviations

DPN	DOX-loaded PLGA nanoparticle
iDPN	PEI was added in W1 during the incorporation process
i’DPN	PEI was added in O during the incorporation process
IDPN	PEI-DPN
AIDPN	PAA-PEI-DPN
